# Photodegradation Kinetics and Solvent Effect of New Brominated Flame Retardants (NBFRS) in Liquid Medium

**DOI:** 10.3390/ijerph191811690

**Published:** 2022-09-16

**Authors:** Yan Lv, Jun Jin, Ru Li, Ruiwen Ma, Weixiang Huang, Ying Wang

**Affiliations:** 1 College of Life and Environmental Sciences, Minzu University of China, Beijing 100081, China; 2 Beijing Engineering Research Center of Food Environment and Public Health, Minzu University of China, Beijing 100081, China

**Keywords:** new brominated flame retardants, solvent effect, density functional calculation, reactive site, degradation pathway

## Abstract

**Highlights:**

Effects of different lights, concentrations and solvents on the degradation kinetics of NBFRs; energy changes in electron transfer are the key factor influencing solvent effects; prediction of degradation pathways and reactive active sites of NBFRs.

**Abstract:**

The photolysis of four typical NBFRs, hexabromobenzene (HBB), pentabromotoluene (PBT), pentabromobenzyl acrylateare (PBBA) and pentabromoethylbenzene (PBEB), were explored under different irradiation light wavelengths, initial concentrations and organic solvents. Density functional theory was used for chemical calculation to explore the internal mechanism of solvent effect. All degradation kinetics conformed to the first-order kinetic model. Under different irradiation light wavelengths, the degradation rates were in the following order: 180~400 nm (0.1702~0.3008 min^−1^) > 334~365 nm (0.0265~0.0433 min^−1^) > 400~700 nm (0.0058~0.0099 min^−1^). When the initial concentration varied from 0.25 mg/L to 1 mg/L, the degradation rate decreased from 0.0379~0.0784 min^−1^ to 0.0265~0.0433 min^−1^ under 334~365 nm irradiation, which might be attributed to the reduction in light energy received per unit area and competition from intermediate metabolites. In different organic solvents, the degradation rates were in the order of acetone (0.1702~0.3008 min^−1^) > toluene (0.0408~0.0534 min^−1^) > n-hexane (0.0124~0.0299 min^−1^). Quantum chemical calculation and analysis showed that the energy change in electron transfer between solvent and NBFRs was the key factor to solvent effect in the degradation of NBFRs. The active sites and degradation pathways of NBFRs were also speculated, the nucleophilic reaction of the Br atom on a benzene ring was the main process of photodegradation and it was preferential to remove the bromine and then the ethyl group on the benzene ring. Our research will be helpful in predicting and evaluating their photochemical behavior in different environment conditions.

## 1. Introduction

Brominated flame retardants (BFRs) are a kind of important flame retardants, which are widely used in furniture, textiles, carpets, electronic castings, automobile parts, building materials, insulators and other consumer goods to improve the flame retardancy of products due to their good flame retardancy [1,2]. However, BFRs are gradually becoming restricted or prohibited in some countries or in international legislation due to their environmental accumulation and toxicity [3,4]. For example, European Union regulations have banned polybrominated biphenyls, pentabromodiphenyl ether, octabromodiphenyl ether and decabromodiphenyl ether. In this case, new brominated flame retardants (NBFRs) and polymer brominated flame retardants (PBFRs) [5,6] began to be produced and used in large quantities as their substitutes.

The demand for new brominated flame retardants, as a substitute for the discontinued traditional brominated flame retardants, is increasing in the consumer market year by year. Some scholars estimate that the global output of NBFRs is estimated to be about 100,000–180,000 tons per year [7]. In recent years, with the mass production and use of NBFRs, they have been widely present in various environmental media, even human blood [8,9]. Hexabromobenzene (HBB), pentabromotoluene (PBT), pentabromobenzyl acrylateare (PBBA) and pentabromoethylbenzene (PBEB) are considered as four important currently used NBFRs with respect to production and consumption all over the world [10,11,12,13,14,15,16]. The concentration levels of HBB, PBEB and PBT in the atmospheric environment has shown an upward trend [17]. Previous studies have shown that NBFRs are persistent, easy to bioaccumulate and highly toxic [18]. Moreover, PBEB has been included in the OSPAR list of priority controlled chemicals in the EU [10]. Studies have shown that PBT and PBBA have biomagnification effects, and the trophic amplification factors of PBT and PBBA are 4.5 and 4.6, respectively [19]. It is noteworthy that NBFRs have also been found in the Arctic region [20], proving that NBFRs can exist in the environment for a long time and be transported over long distances and are a typical type of persistent organic pollutants (POPs).

Photodegradation is an efficient and energy-saving method to remove organic pollutants from the environment. Photodegradation has more efficient performance compared with microbial degradation with slow degradation rate [20,21]. At present, some researchers have conducted preliminary studies on the photodegradation of NBFRs, mainly from two aspects: on the one hand, the effects of experimental conditions, including light conditions, environmental media [22,23] and the degradation kinetics of NBFRs [24,25]; on the other hand, the degradation pathway and degradation mechanism of NBFRs [26,27]. It can be found that organic solvents can be used to simulate the composition of environmental media [28], and research on the transformation of NBFRs in different polar solvents is conducive to analyzing their degradation characteristics in the actual environment. At the same time, chemical calculation can help researchers find mechanisms from a micro perspective [29]. Davis et al. [24] and Wang et al. [25] studied the photodegradation of NBFRs in different organic solvents. Their experimental results showed that the half-life of NBFRs was affected by the solvent medium. Jiang et al. [30] researched the effect and promotion mechanism of solvent molecules on the photodegradation of PBDEs with DFT method considering the solvent effect. Chen et al. found that the substitution pattern for chlorine atoms, the dipole moment, and ELUMO-EHOMO were major factors in the photolysis of PCPDSs through experiments and calculations. However, the mechanism of the effect of solvents on the photodegradation of NBFRs has been rarely studied.

At present, there is no unified conclusion on the degradation properties and persistence of NBFRs. Therefore, the study of their degradation kinetics and half-life needs to be further carried out. In this study, the degradation kinetics and half-life of HBB, PBT, PBEB and PBBA were studied under different wavelengths of light, initial concentrations and solvents, density functional theory was used for chemical calculation to explore the effect of solvent on the photodegradation mechanism. At the same time, combined with theoretical calculation and the GC-MS mass spectrum of degradation products, the active sites and degradation pathways of NBFRs were speculated. 

## 2. Materials and Methods

### 2.1. Chemicals and Materials

All solvents used in the experiment were of high-performance liquid chromatography (HPLC) grade. Acetone, dichloromethane and n-hexane were obtained from J.T. Baker (Phillipsburg, NJ, USA) and purchased from Adamas (Shanghai, China). Toluene was obtained from Duksan Pure Chemicals Co., Ltd. (Seoul, Korea), and ultra-pure water was produced using a Milli-Q system (EMD Millipore, Billerica, MA, USA). Other chemical reagents used in the experiment were all analytically pure at the least. PBBA and PBT were obtained from AccuStandard (New Haven, CT, USA). A PBEB standard was purchased from Dr. Ehrenstorfer (Augsburg, Germany). An HBB standard was purchased from Tokyo Chemical Industry (Shanghai, China).

Amounts of 50 mg/L individual stock solutions of HBB, PBEB, PBT and PBBA were prepared by dissolving solid compounds into acetone. An appropriate amount of stock solution was then taken and gradually diluted with different organic solutions (n-hexane, toluene and acetone) into a final concentration of 1 mg/L for further photodegradation experiments.

### 2.2. Irradiation Experiments

The photodegradation experiments were carried out by LAB500E436800 multi-photochemical reactor, equipped with a merry-go-round apparatus and a Teflon-coated magnetic stirrer to ensure uniform light exposure and complete mixing of all solutions and the ultraviolet and visible lamps. Multi-photochemical reaction instruments and supporting light source systems were purchased from Beijing Zhongjiao Jinyuan Technology Co., Ltd. (Beijing, China). The light intensity in the sample region was 125 mW/cm^2^, measured by an ultraviolet radiometer. A schematic diagram of the reactor was given as Supplementary Material (Figure S3). All photodegradation experiments were carried out under the condition of continuous circulating water to achieve a constant temperature of 15 °C. The chiller providing constant temperature circulating water was purchased from Zhengzhou Changcheng science industry and Trade Co., Ltd. (Zhengzhou, China).

The light source for the light reaction was provided by a 500 W mercury lamp and a xenon lamp including three wavelength ranges, namely: 180~400 nm, 334~365 nm, 400~700 nm. Thirty milliliters of NBFRs solutions were prepared for radiation experiments, and the pH was not adjusted in typical runs. PBBA, PBEB, PBT and HBB in n-hexane, toluene and acetone were irradiated under 180~400 nm at the initial concentration of 1 ppm. PBBA, PBEB, PBT and HBB in 0.25 mg/L, 0.5 mg/L and 1 mg/L were irradiated under 334~365 nm and 180~400 nm in the n-hexane. Samples were collected at specific time points: 0/1/2/5/10/20/30/45/60 min for 180~400 nm and 334~365 nm and 0/1/2/5/10/20/30/60/90/120 min for 400~700 nm. Solvent blank and dark control samples were performed for each treatment, and all treatments were conducted in triplicate.

### 2.3. Instrumental Analysis

The extracts were quantitatively analyzed using an Agilent 6890 gas chromatograph (Agilent Technologies, Santa Clara, CA, USA). The mass spectrometer was used in negative chemical ionization mode and selected ion monitoring mode. The carrier gas was helium (1.0 mL/min), and the reagent gas was methane (1.0 mL/min). The temperatures of the injector, mass spectrometer source and quadrupole were 290 °C, 150 °C and 150 °C respectively.

The PBBA, PBT, PBEB and HBB were analyzed using a different J&W DB-5 MS column (30 m long, 0.25 mm i.d., 0.1 mm film thickness; Agilent Technologies). The oven temperature program started at 100 °C, which was held for 3 min, then increased at 4 °C/min to 300 °C, which was held for 8 min. The fullscan of PBBA, PBT, PBEB and HBB were analyzed under the temperature program that the oven temperature started at 45 °C, increased at 15 °C/min to 200 °C, then increased at 6 °C/min to 300 °C, which was held for 5 min.

The *m*/*z* ratios 485.6 and 487.6 were monitored for PBBA and PBT. The *m*/*z* ratios that were monitored for PBEB and HBB were 499.6 and 501.6, 547.6 and 549.6, respectively.

### 2.4. Quantum Chemical Calculation

The quantum chemical calculations were constructed in Gauss view 6.0 and then performed by Gaussian 16 W with the Density Functional Theory (DFT) method [30,31,32]. The optimal structures of PBBA, PBEB, HBB and PBT in the ground state were first calculated at the B3LYP/6-31g(d) level of theory via the density functional theory (DFT). The subsequent operation would be carried out at the B3LYP/6-31g(d) level on the basis of the ground state optimal structure. The excited state energy of NBRFs were calculated by the time-dependent density functional theory (TD-DFT), which gave the vertical transition energy (E_T1_, eV). The vertical ionization energy (VIE, eV) and vertical electron affinity energy (VEA, eV) of NBRFs were calculated by DFT. At the same time, the effects of solvents on molecular structure and molecular frontier orbitals were calculated by DFT in order to analyze the effects of solvents on the photodegradation of NBFRs at the molecular level. Solvation effects were considered in the above solvation calculations and the polarisable continuum model (PCM) [33] of self-consistent reaction field (SCRF) was adopted. PCM is one of the most widely used SCRF models because its theoretical values were highly consistent with the experimental values [34,35].

### 2.5. Study on Degradation Solvent Effect

**Figure 1:**
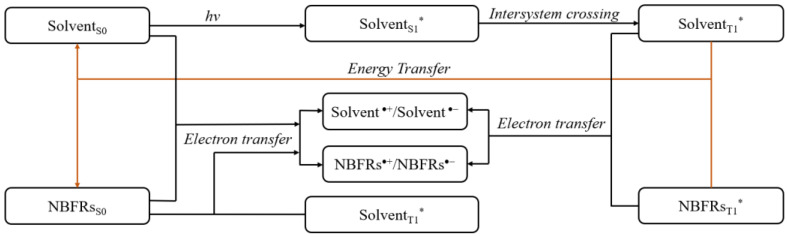
Photoinduced energy/electron transfer pathways between solvent molecules and NBFRs. * Represents the excited triplet state of the molecule.

Solvent molecules may interact with NBFR molecules through energy transfer or electron transfer [36,37]. Taking excited triplet states as an example, the reaction path of energy/electron transfer between solvent molecules and NBFRs is shown in the Figure 1. The Gibbs energy variation (ΔG) of the redox reaction can be calculated from the oxidation potential of the electron donor (VIE_D_) and the reduction potential of the electron acceptor (VEA_A_) in the reaction, so ΔG = VIE_D_ − VEA_A_ [38,39]. If the electron do nor or acceptor is in an excited state, the vertical transition energy of the electron donor or acceptor should also be considered when calculating the ΔG.

Solvent_T1_* sensitizes NBFRs_S0_ to NBFRs_T1_* by energy transfer: Solvent_T1_* + NBFRs_S0_→ NBFRs_T1_* + Solvent_S0_, where S0 represents the ground state and T1 represents the first excited triaxial state. The reactivity of the light-induced reaction between NBFRs and solvent molecules was judged by comparing the vertical transition energy of Solvent_T1_^*^ and NBFRs_T1_* [40]. The subsequent light-induced reactions between NBFRs and solvent molecules are listed in Table 1. 

Vertical ionization energy (VIE) and vertical electron affinity potential energy (VEA) need to be calculated on the basis of stable material structure, the calculation methods are as follows:

VIE_S0_ = E (M + 1) − E (M0)

VEA_S0_ = E (M) − E (M-1)

VIE_T1_ = VIE_S0_ − E (T1)

VEA_T1_ = VIE_S0_ + E (T1)

## 3. Results and Discussion

### 3.1. Degradation under Different Light Wavelength

**Figure 2:**
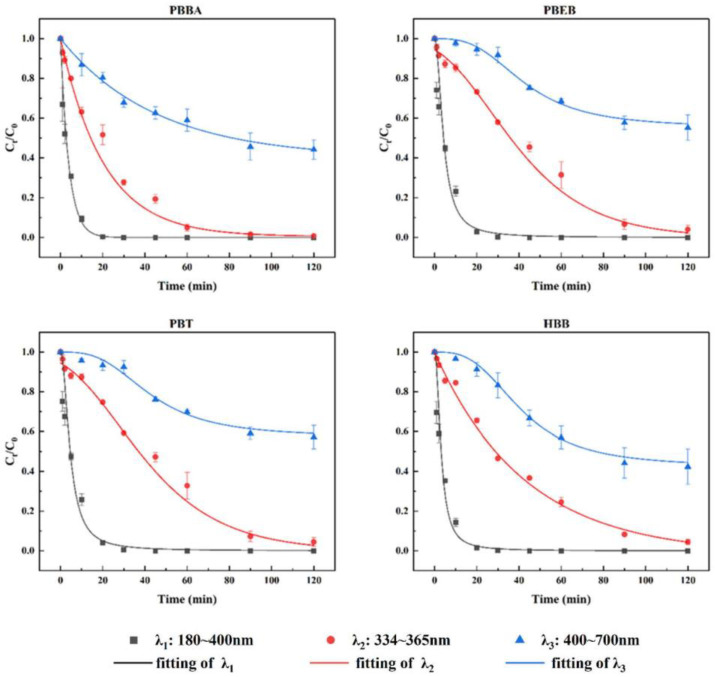
The degradation of PBBA, PBEB, PBT and HBB under three different wavelengths (λ_1_: 180~400 nm, λ_2_: 334~465 nm, λ_3_: 400~700 nm). The initial concentration was 1 mg/L. The reaction temperature was 15 °C.

The degradation kinetics of PBBA, PBEB, PBT or HBB in n-hexane was conducted under three different wavelengths (20 °C). The wavelength ranges of λ_1_, λ_2_ and λ_3_ were 180~400 nm, 334~365 nm and 400~700 nm, respectively. No target NBFRs were detected in their blank samples. None of dark control samples degraded. As shown in Figure 2, all NBFRs degraded under the irradiation of three different wavelengths because we observed a clear concentration decline of NBFRs and a clear increase sign of degradation products. The linear fit between ln C_t_/C_0_ and t was good (R^2^ > 0.95), indicating that the photodegradation of NBFRs under three light conditions followed quasi-first-order kinetics. C_t_ and C_0_ represent the concentration of NBFRs at t and 0 min, respectively. 

In this study, the half-life refers to the time taken for the content of NBFRs to reduce to half of the initial value by light reaction, and the half-life can reflect the environmental persistence of the substance. By fitting the photodegradation data to pseudo-first-order kinetics equation, the half-life among three wavelengths (λ_1_, λ_2_ and λ_3_) were 2.31–71.93 min for PBBA, 3.57–112.12 min for PBEB, 3.85–120.40 min for PBT and 4.07–79.93 min for HBB, respectively. The specific degradation rate constant and half-life are shown in Table 2. The half-life of the four NBFRs showed a common tendency as λ_3_ > λ_2_ > λ_1_. The degradation rate of PBBA, PBEB, PBT or HBB under 180~400 nm was 18.7~30.8 times that under 400~700 nm, while the degradation rate under 334~365 nm was 2.9~4.8 times that under 400~700 nm, which showed that the wavelength of 180~334 nm was the main contribution band for the photodegradation of PBBA, PBEB, PBT and HBB, which was consistent with the previous research [26]. 

The calculated half-life results show that the half-life of NBFRs has a certain regularity under different wavelengths. The order of the half-lives are shown as HBB > PBT > PBEB > PBBA under the UV light, which includes wavelengths of λ_1_ and λ_2_. However, the half-lives are shows as PBT > PBEB > HBB > PBBA under the Visible light, that is, λ_3_. The increase in the half-life of HBB is not as large as that of PBT or PBEB with the increase in light source wavelength. This may be related to the wide light energy absorption band of HBB between 200 and 351 nm, which can be obtained from the ultraviolet visible absorption spectrum in the HBB structure optimization calculation results, as shown in the absorption spectra in Supplementary Material (Figure S1). 

### 3.2. Effect of Initial Concentration on Photodegradation

The initial concentration could be an important parameter in the degradation process. When using photodegradation to degrade NBFRs in environmental media, the degradation time should be considered according to different concentrations. The photolysis of NBFRs was carried out under two kinds of wavelength ranges with three initial NBFRs concentrations ranging from 0.25 mg/L to 1 mg/L, and the above two light sources with different wavelengths and energies could reflect the basic degradation law of NBFRs. The reactions were fitted well by the pseudo-first-order kinetics model. The specific degradation rate constant and half-life are shown in Table 3 and Table 4.

**Figure 3:**
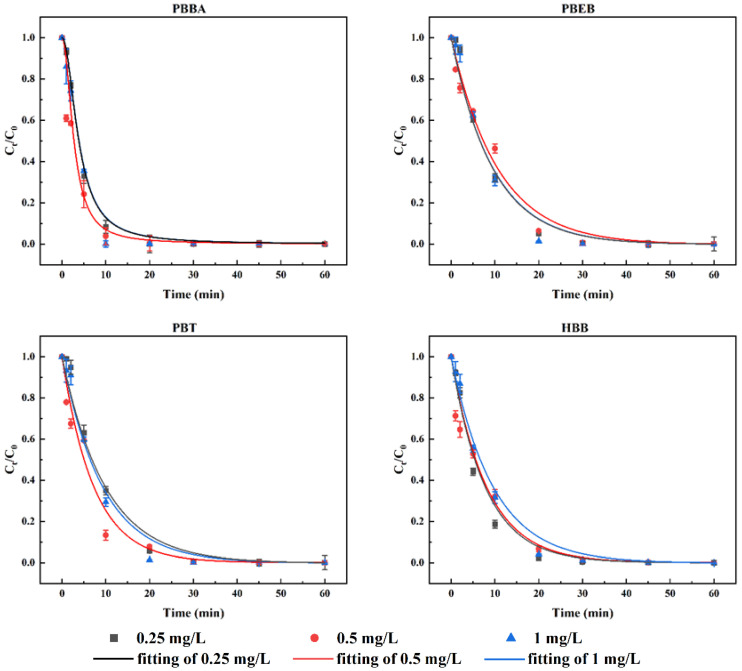
The degradation of PBBA, PBEB, PBT and HBB with three different initial concentrations (0.25 mg/L, 0.5 mg/L, 1 mg/L) under light wavelength of 180~400 nm. The reaction temperature was 15 °C.

The degradation rate of NBFRs under light wavelength of 180~400 nm is basically not affected by the initial concentration. When the initial concentration is increased from 0.25 mg/L to 1 mg/L, the degradation percentage of PBT, PBEB, HBB can reach 99% in 30 min and that of PBBA can reach 99% in 20 min (Figure 3). 

**Figure 4:**
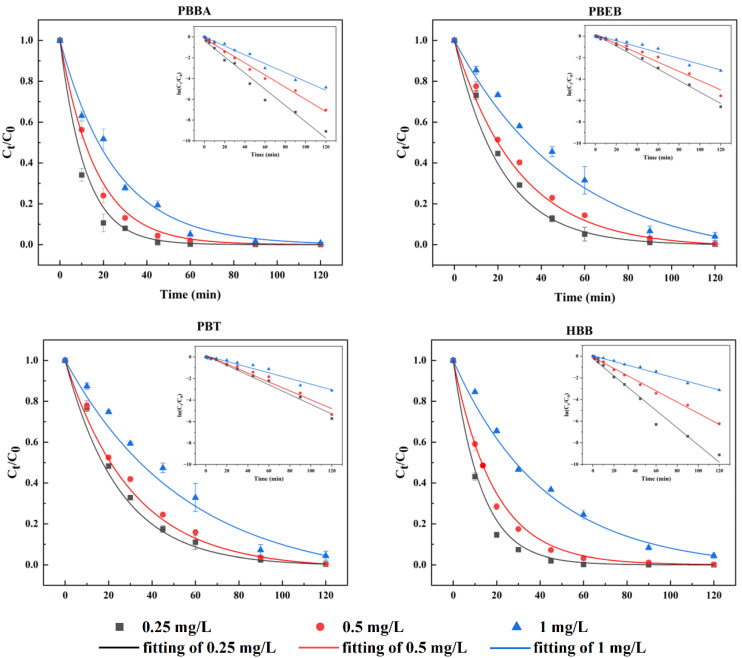
The degradation of PBBA, PBEB, PBT and HBB with three different initial concentrations (0.25 mg/L, 0.5 mg/L, 1 mg/L) under light wavelength of 334~365 nm. The reaction temperature was 15 °C.

The degradation rate of NBFRs under light wavelength of 334~365 nm showed a downward trend with the increase in the initial concentration (Figure 4). NBFRs at three initial concentrations can be basically completely degraded at 120 min under the wavelength of 334~365 nm. However, as the concentration of NBFRs increased from 0.25 mg/L to 1 mg/L, the degradation rate of NBFRs decreased at 60 min when the reaction time reached 60 min. 

On the one hand, the photolysis rate was positively correlated with the light energy received by NBFRs molecules per unit area. When the irradiated light energy is constant, the higher concentration of NBFRs, the less light energy per NBFRs molecule. Meanwhile, the amounts of activated radicals (OH or O) were insufficient for the oxidation of high concentration NBFRs [41]. On the other hand, NBFRs produce many intermediate metabolites in the process of photodegradation. They can compete with NBFRs for photons, which reduces the quantum efficiency of NBFRs to absorb photons and affects its photodegradation rate [42,43,44].

### 3.3. Degradation in Different Organic Solvents under UV Exposure

**Figure 5:**
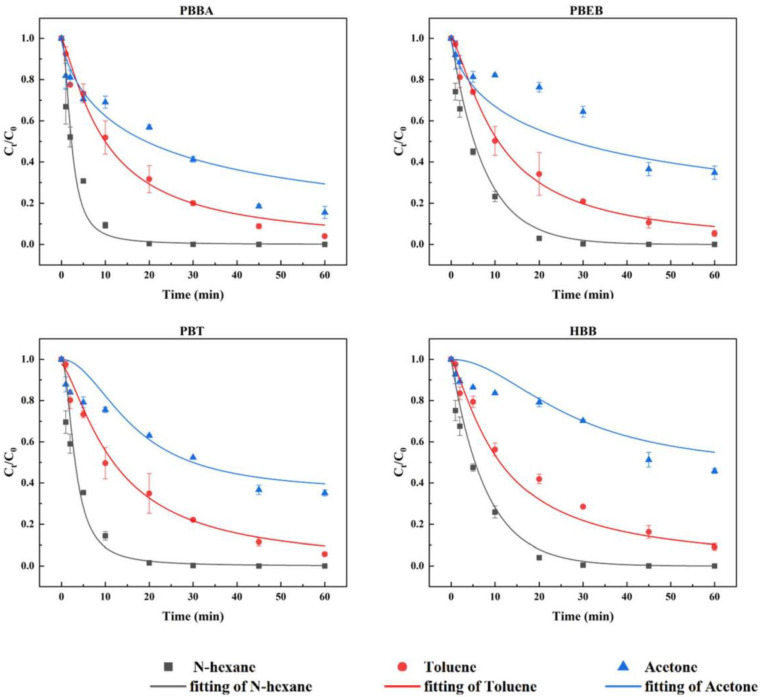
The degradation of PBBA, PBEB, PBT and HBB in three different solvents (n-hexane, toluene, acetone) under light wavelengths of 180~400 nm. The initial concentration was 1 mg/L. The reaction temperature was 15 °C.

In view of the complex and changeable components of environmental media, organic solvents can be used to simulate the components of environmental media, such as soil environments with different polarities, to explore the photochemical behavior of pollutants in environmental media. The photolysis of NBFRs was carried out under a 180~400 nm wavelength of a 500 W mercury lamp with three organic solvents (n-hexane, toluene and acetone). As shown in Figure 5, the reactions were fitted well by the pseudo-first-order kinetics model. The degradation rate constant, half-life and correlation coefficient of the fitting curve are given in Table 5.

The degradation efficiencies of NBFRs were significantly affected by the type of solvent, which is consistent with the previous works that the rate coefficients of PBDEs, DBDPE and PAHs were greatly affected by organic solvents [45,46,47,48]. The degradation percentage of NBFRs in n-hexane can basically reach 99% when exposed to light for 30 min. However, NBFRs in toluene and acetone did not achieve degradation equilibrium within 60 min. The half-life of NBFRs in n-hexane, toluene and acetone were 2.31~4.07 min, 13.00~17.02 min and 23.19~56.15 min, respectively. A similar half-life trend for NBFRs in solvents was observed: n-hexane < toluene < acetone.

The cut-off wavelengths of n-hexane, toluene and acetone are 200 nm, 285 nm and 330 nm, respectively, indicating that the absorption capacity of the three solvents to ultraviolet light is increasing; therefore, their competition with NBFRs molecules to absorb light energy is increasing. This is one of the reasons for the decreasing photodegradation rate of NBFRs in n-hexane, toluene and acetone. The deeper reason is the effect of solvent molecules on NBFRs molecules, resulting in changes in the NBFRs’ molecular configuration [49], orbital energy difference, molecular dipole moment and electron transfer reaction. A detailed analysis is provided in the theoretical study on solvent effects in the next section.

### 3.4. Theoretical Study on Solvent Effect

#### 3.4.1. Effect of Solvent on Molecular Structure of NBFRs

The effect of solvents on the molecular structure of NBFRs was analyzed from two aspects: molecular chemical bond length and molecular dipole moment. Specific data are given in Supplementary Material (Table S1).

**Figure 6:**
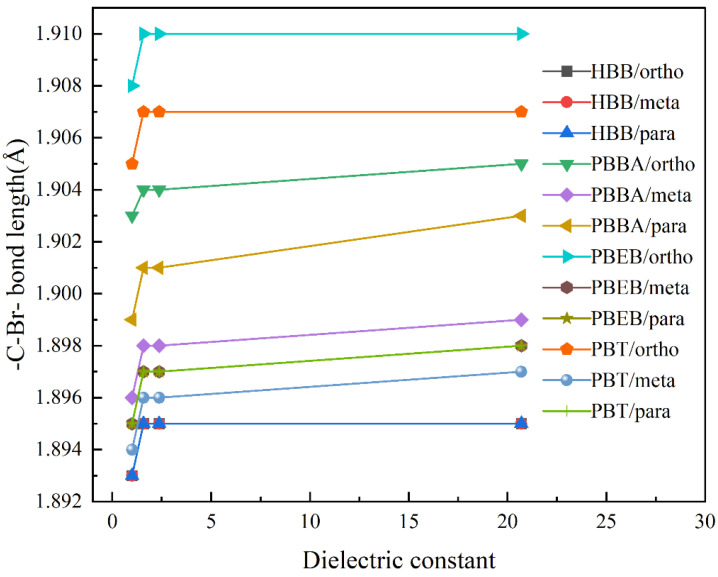
Change curve of C–Br bond lengths of NBFRs with the dielectric constant.

The molecular optimization results display that the C–C bond length of the benzene ring’s substitution position in PBBA, PBEB and PBT molecules gradually decreases with the increase in solvent polarity, and the decreasing trend is gradually flat. As shown in Figure 6, the C–Br bond lengths of NBFRs show an increasing trend with the increase in the solvent dielectric constant, which tends to gradually become flat. From the perspective of the molecular structure, the longer the bond between two atoms in a molecule, the smaller their bond energy, and the greater the activity of the chemical reaction at this position. In conclusion, the greater the polarity of the NBFR reaction medium solvent, the lower the chemical reaction activity of the benzene ring substitution position in the NBFR molecule, and the more difficult it is to break the C–C bond of the benzene ring substituent. For NBFRs with substituents on the benzene ring, the C–Br bond at the ortho position has high reactivity and is prone to fracture, followed by the para position and, finally, the meta position. Moreover, with the increase in the solvent polarity of the reaction medium, the reaction activity of the C–Br bond on the benzene ring increases to varying degrees. PBBA is most affected by solvent polarity. 

**Figure 7:**
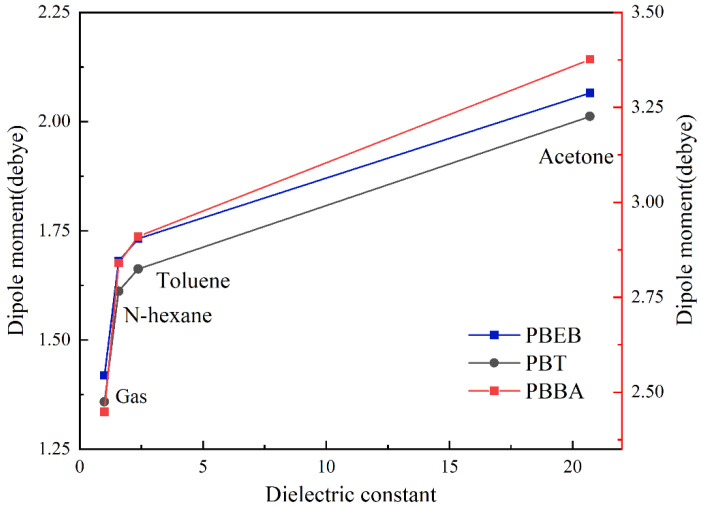
The relationship between dipole moments of NBFRs (PBEB, PBT, PBBA) and dielectric constants.

In addition, the dipole moment of NBFRs molecules in different solvents also changed. As shown in Figure 7, when the solvent changed from gas to acetonitrile, the dielectric constant of the solvent increased and the dipole moment, which was the polarity of the PBBA, PBEB and PBT molecules, gradually raised with the increase in the polarity of the solvent. However, the increase amplitude decreased, indicating that the effect of the solvent tends to be saturated. The dipole moment of HBB was always 0 due to its central symmetry, so it was not considered in this part. It could be seen that the dipole moment and polarity of NBFRs would increase with the increase in solvent polarity, but there was a phenomenon of solvent effect saturation.

#### 3.4.2. Effect of Solvents on Frontier Orbitals of NBFRs

The ΔE_gap_ between LUMO and HOMO orbitals can reflect the stability of NBFRs molecules, as shown in Table 6. It can be seen that different solvents will affect the E_HOMO_ and E_LUMO_ of NBFRs molecules, and the ΔE_gap_ of NBFRs decreased with the increase in the solvent dielectric constant [50], indicating they were more prone to chemical reaction, which was inconsistent with the experimental results. The above calculation results showed that the influence of solvent on the orbital energy levels of NBFRs was not the dominant factor in the final photodegradation results. 

#### 3.4.3. Properties of Solvent Excited States and Its Influence Mechanism on the Photolysis of NBFRs

The energy parameters (E_T1_, VIE and VEA) involved in induced reactions between NBFRs and solvent molecules are presented in Table 7. N-hexane cannot effectively absorb the light from the UV light source because its cut-off wavelength is 220 nm, which is less than the UV absorption wavelengths of NBFRs in n-hexane [51]. For the three solvents of n-hexane, acetone and toluene, the value of E_T1_ (Solvent) is larger than E_T1_ (NBFRs), indicating that the excited solvent molecules can sensitize the NBFRs from ground state to excited state via energy transfer: NBFRs_S0_ + Solvent_T1_* → NBFRs_T1_* +Solvent_S0_, which corresponds to the requirements for the light-induced reaction. NBFRs_T1_* generated by the reaction may continue to react with Solvent_T1_* or Solvent_S0_. The energy changes in ΔG for subsequent reactions are shown in Table 8.

The ΔG1 and ΔG2 of the photoinduced reaction 1 and reaction 2 are positive, indicating that the photoinduced reaction between NBFRs_T1_^*^ and Solvent_S0_ cannot occur spontaneously because the E_T1_ of each solvent is larger than that of NBFRs. 

The ΔG3 and ΔG4 of the photoinduced reaction 3 and reaction 4 are negative, indicating that not only the photoinduced reaction between NBFRs_T1_^*^ and Solvent_T1_^*^ can occur spontaneously, but the energy transfer is a continuous process. At the same time, it is not difficult to find that the continuous energy transfer process from excited solvent molecules to NBFRs molecules is effective not only for their ground states but also for their excited states by comprehensively analyzing the four reaction processes from reaction 1 to reaction 4. Meanwhile, the calculated ΔG3 and ΔG4 of NBFRs in n-hexane are the lowest among those in these three solvents. The E_T1_ of NBFRs were also the smallest in n-hexane Table 7, implying that reaction between Solvent_T1_^*^ and NBFRs_T1_^*^ are the main factor to promote NBFRs photodegradation, which is consistent with the previous experimental results. In the photodegradation reaction of NBFRs in toluene, ΔG3 is negative; however, ΔG4 is positive, indicating that reaction 3 is the main reaction pathway, where Solvent_T1_^*^, as an electron donor, reacts with NBFRs as electron acceptors. According to the theoretical calculation, the photodegradation efficiency in toluene should be the lowest instead of the experimental results that the photodegradation effect in acetone is the worst. Considering that a benzene ring is a conjugated system with low electronegativity and stronger electron acceptance ability, the calculated VEA_S0_ of toluene is less than 0, which also verifies this point, indicating that the strong electron obtaining ability of toluene plays a leading role in the photodegradation of NBFRs in toluene.

The calculated ΔG5 and ΔG6 of NBFRs in toluene and acetone are positive, demonstrating the reaction between Solvent_T1_* and NBFRs_S0_ cannot happen. This theoretical calculation result coincides with the results of their inhibition effects on the photodegradation of NBFRs. The ΔG6 values of NBFRs in n-hexane were negative, indicating the photodegradation of NBFRs in n-hexane was spontaneous, which can be explained from two aspects: On the one hand, the reaction is affected by the electron ionization ability of ground state NBFRs molecules [52]. The vertical ionization energy of different ground state NBFRs molecules is different. The larger the VIE of ground state molecules, the easier it is to provide electrons. The calculation results show that the VIE of NBFRs_S0_ molecules in n-hexane are the largest among the three solvents in the experiment, as shown in Table 7, which is consistent with the result of the fastest photodegradation rate of NBFRs in n-hexane. On the other hand, the reaction is also affected by the electron acquisition ability of excited-state solvent molecules [53]. The higher the electron affinity energy of excited-state solvent molecules, the stronger its ability to obtain electrons, which is more conducive to the formation of free radical cations of NBFRs, so as to promote the photodegradation of NBFRs. The electron affinity energies of excited-state n-hexane molecules are greater than those of other solvents, so n-hexane has stronger electron acquisition ability, which makes the photodegradation of NBFRs in n-hexane is more likely to occur.

### 3.5. Reactive Site Prediction

**Figure 8:**
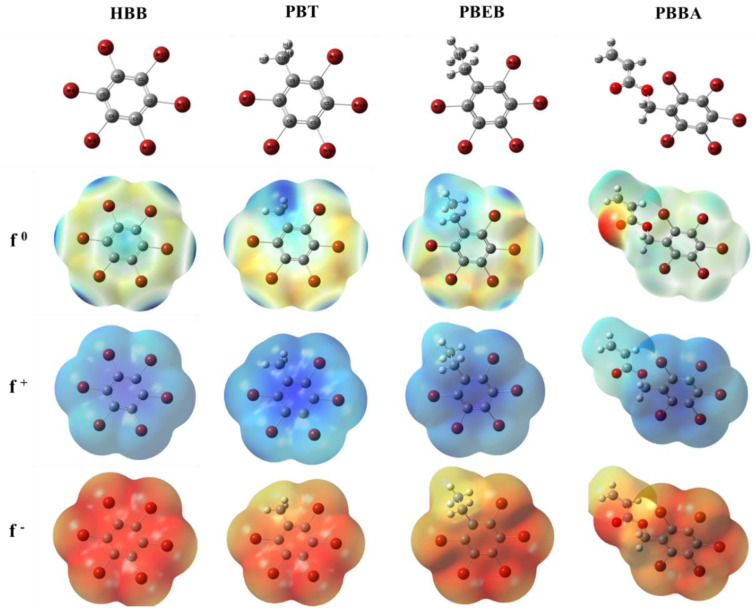
Molecular structure diagram (marked with atomic position number) and the Fukui function mapped electron density isosurface (ρ = 0.01 a.u.): *f*^0^ (*r*), *f*^+^ (*r*), and *f*^−^ (*r*) of HBB, PBT, PBEB, and PBBA (the dark blue on the isosurface denotes the larger positive value of the Fukui function).

The Fukui function can be used to predict the degradation active sites of NBFRs. The Fukui-function-mapped electron density isosurface was plotted using GaussView, as shown in Figure 8. For the Fukui function, the region with a larger positive value is more likely to be the active site of the corresponding reaction, corresponding to the dark blue region. *f*^0^, *f*^+^, and *f*^−^ were performed for predicting the radical attack sites, nucleophilic attack sites, and electrophilic attack sites, respectively [54].

In the electron density isosurface of HBB, the most positive values of *f*^0^ occur at C1/2/3/4/5/6 > Br7/8/9/10/11/12, which are the sites with the highest probability of radical attack. The most positive values of *f*^+^ occur at Br7/8/10/12 > Br9/11 > C2/3/5/6 > C1/4, which are the sites with the highest probability of nucleophilic attack. The most positive values of *f*^−^occur at C1/2/3/4/5/6 > Br7/8/9/10/11/12, which are the sites with the highest probability of electrophilic attack. 

In the electron density isosurface of PBT, the atomic order of positive electrostatic potential values in *f*^0^ is H13~15 > C6 > C2/4 > C3 > C1 > C5 > Br9; the atomic order of positive electrostatic potential values in *f***^+^** is H13~15 > C6 > Br12 > Br8 > Br7 > Br11 > Br9; and the atomic order of positive electrostatic potential values in *f*^−^ is H13~15 > C6 > C2/4 > C3 > C5 > C1 > Br12.

In the electron density isosurface of PBEB, the atomic order of positive electrostatic potential values in *f*^0^ is H14~18 > C6 > C2/4 > C3 > C1/5 > Br9 > Br8/12; the atomic order of positive electrostatic potential values in *f***^+^** is H14~18 > C6 > Br8/12 > Br7/11 > C2 > C4 > Br9; and the atomic order of positive electrostatic potential values in *f*^−^ is H14~18 > C6 > C4 > C2 > C3 > C1/5 > Br8/12.

In the electron density isosurface of PBBA, the atomic order of positive electrostatic potential values in *f*^0^ is C14 > H18~22 > C5 > C1 > C3 > C2 > C6; the atomic order of positive electrostatic potential values in *f***^+^** is C14 > H18~22 > Br8 > Br10 > Br7 > C5 > Br11; and the atomic order of positive electrostatic potential values in *f*^−^ is C14 > H18~22 > C5 > C2 > C1 > C3 > C6.

In general, C atoms at the benzene ring substitution position of NBFRs have strong reactivity [55]. H atoms in the substituted group of the benzene ring have strong reactivity; however, C atoms in the substituted group of the benzene ring have the weakest reactivity. The results also show that the Br atoms connected to the benzene ring are strong nucleophilic reaction sites. Combined with the degradation pathway of NBFRs, the nucleophilic reaction of Br atom on benzene ring is the main process of photodegradation.

### 3.6. Degradation Pathway

**Figure 9:**
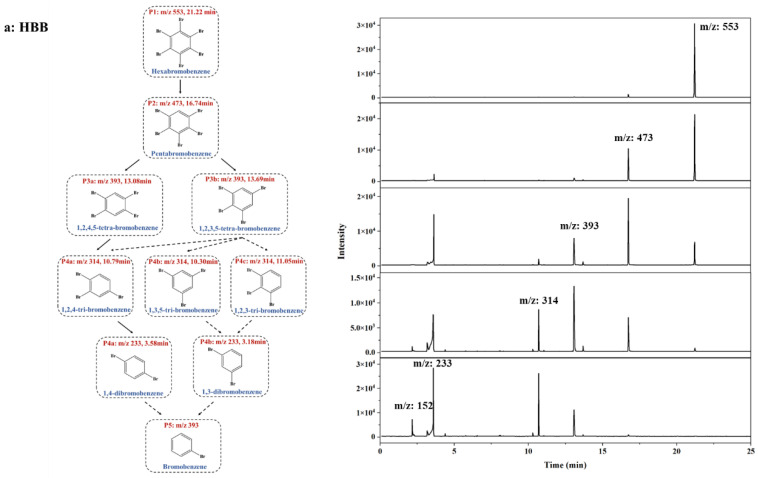

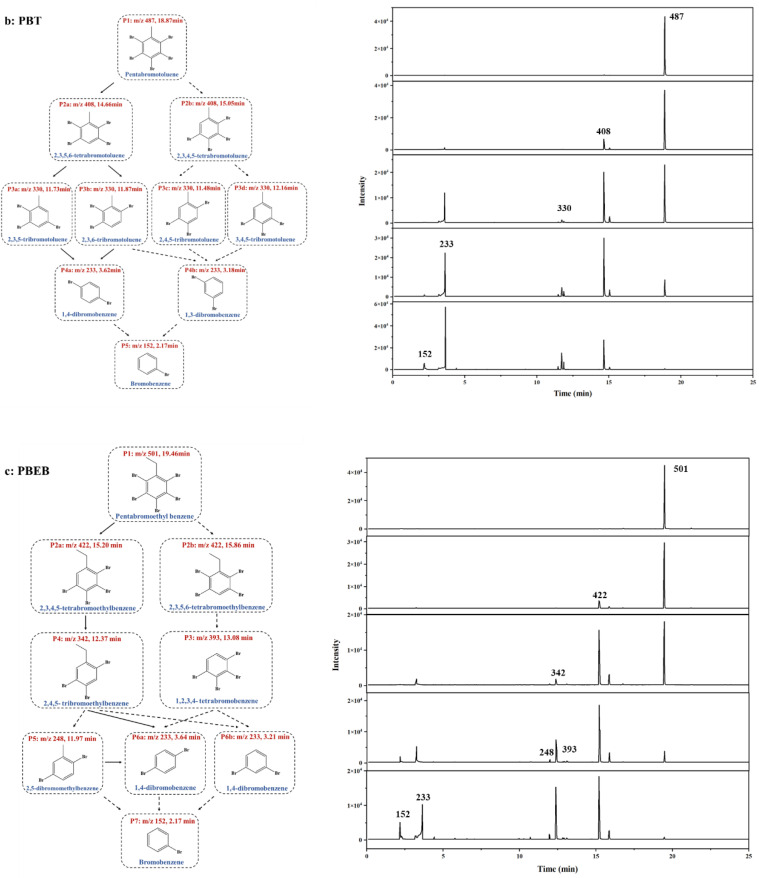

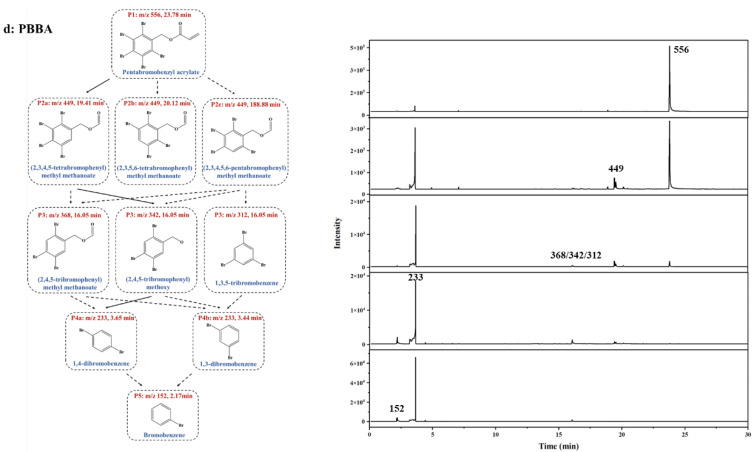
Proposed photolytic pathways and mass spectra of (**a**) HBB, (**b**) PBT, (**c**) PBEB, (**d**) PBBA. The solid arrow indicates the primary degradation pathway, and the dotted line indicates the minor pathway. (In the same group of peaks, the peaks with the highest proportion of peak area to the lowest were labeled a, b, c, and so on.) The mass spectrum of each substance from bottom to top is 0/2/10/30/60 min of photodegradation reaction.

The research on the degradation pathway of NBFRs plays an important role in their pollution control process. The study of the degradation pathway can accurately judge the types and generation time of possible metabolites in the treatment of NBFRs by light, so that reasonable conditions can be selected to make the photodegradation of NBFRs proceed in the desired direction. The degradation product experiment of NBFRs were carried out under the wavelength of 180~400 nm exposure and n-hexane as the solvent. Take the sample at 0/2/10/30/60 min during degradation as an example and conduct full scanning under the CI source. The mass charge ratio of the peaks, whose abundance were coherently changing in the mass spectrum, were marked and analyzed. At the same time, the energy and stability of the possible debromination intermediates and degradation products of NBFRs were calculated, and the reaction active sites of NBFRs were predicted in Section 3.5. Based on the above, the mass spectra and degradation pathways of NBFRs are shown in Figure 9. 

In the mass spectrometry of HBB, the peaks with the same mass charge ratio are grouped into the same group. The six groups of peaks were numbered successively according to the mass charge ratio from largest to smallest, which is 553, 473, 393, 314, 233 and 152. In the degradation process, hexabromobenzene is gradually de-brominated and can finally be degraded into dibromobenzene and monocromobenzene, but mainly dibromobenzene, indicating that reductive de-bromination is the main degradation pathway [25,50]. In the degradation process of PBT, bromine is preferentially removed from the benzene ring rather than methyl, which is consistent with the previous calculation that the C–Br bond energy on the benzene ring is lower than the C–C bond energy [56]. The degradation pathway of PBEB is similar to that of PBT, for which it is preferential to remove the bromine and then the ethyl group on the benzene ring. Fragmentations at *m*/*z* of 422 correspond to sequential losses of Br compared with *m*/*z* of 501. Fragmentations at *m*/*z* of 342 and 393 correspond to sequential losses of Br and CH_2_CH_3_, respectively, compared with *m*/*z* of 422. Fragmentations at *m*/*z* of 248 correspond to sequential losses of Br and CH_3_ compared with *m*/*z* of 342. In the degradation process of PBBA, the change from *m*/*z* of 556 to *m*/*z* of 499 correspond to sequential losses of Br and CHCH_2_. Fragmentations at *m*/*z* of 368, 342, and 312 correspond to sequential losses of Br, Br and CO, and Br and CH_2_OCHO, respectively, on the basis of *m*/*z* of 449. PBBA and its intermediate products basically achieved complete degradation in 60 min for PBBA, producing ·OX and ·OH in the degradation process, which can further promote the degradation [57]. 

## 4. Conclusions

Our findings revealed that the degradation rate of NBFRs under 180~400 nm was 18.7~30.8 times that under 400~700 nm, while the degradation rate under 334~365 nm was 2.9~4.8 times that under 400~700 nm, which showed that the wavelength of 180~334 nm can be the main contribution band for the photodegradation of NBFRs. When the initial degradation concentration varied from 0.25 mg/L to 1 mg/L, the effect of concentration on the degradation rate under short wave ultraviolet irradiation could be ignored, and the degradation coefficient of NBFRs under long-wave ultraviolet irradiation decreased from 0.0379~0.0784 min^−1^ to 0.0265~0.0433 min^−^^1^.

The degradation rate of NBFRs in different solvent media was n-hexane > toluene > acetone, which showed that NBFRs may be more easily degraded in non-polar environments. The diversity of the light absorption properties of different solvents and the electron energy transfer between different solvents and NBFRs molecules were the main contributing factors of solvent effect. The calculation results of solvent on NBFRs molecular configuration, frontier orbital energy and electron transfer reaction energy between solvent and NBFRs showed that the change in the electron transfer reaction energy between the solvent and NBFRs was the key factor affecting the degradation rate of NBFRs. Among them, the redox reaction between excited solvent molecules and excited NBFR molecules was the main factor affecting the degradation of NBFRs; the electron affinity potential energy of ground state solvent molecules and the vertical ionization energy of excited solvent molecules also had significant effects. In addition, the absorption properties and the dipole moment of the solvent also contributed to the half-lives of NBFRs. The analysis of electron density isosurface diagram showed that the Br atoms on the benzene ring of NBFRs were more prone to nucleophilic reaction to be removed. Our conducted research will be helpful to predict the environmental persistence of NBFRs and study the degradation of NBFRs under various conditions. Studying the degradation pathway can guide the selection of environmental conditions in the actual photodegradation remediation of NBFRs pollution. The study of the degradation pathway can accurately judge the types and generation time of possible metabolites in the treatment of NBFRs by light, so that reasonable conditions can be selected to make the photodegradation of NBFRs proceed in the desired direction. Revealing the internal mechanism of the effect of different solvents on the photodegradation of NBFRs will be helpful to predict and evaluate their environmental persistence and photochemical behavior in different environmental media. 

## Figures and Tables

**Table 1:** Electron transfer reactions between NBFRs and solvent molecules.

NO.	Reaction Equation	Equation
1	NBFRs_T1_* + Solvent_S0_ → NBFRs·^+^ + Solvent·^−^	ΔG1 = VIE_T1_ (NBFRs) − VEA_S0_ (Solvent)
2	NBFRs_T1_* + Solvent_S0_ → NBFRs·^−^ + Solvent·^+^	ΔG2 = VIE_S0_ (Solvent) − VEA_T1_ (NBFRs)
3	NBFRs_T1_* + Solvent_T1_* → NBFRs·^+^ + Solvent·^−^	ΔG3 = VIE_T1_ (NBFRs) − VEA_T1_ (Solvent)
4	NBFRs_T1_* + Solvent_T1_* → NBFRs·^−^ + Solvent·^+^	ΔG4 = VIE_T1_ (Solvent) − VEA_T1_ (NBFRs)
5	NBFRs_S0_ + Solvent_T1_* → NBFRs·^+^ + Solvent·^−^	ΔG5 = VIE_S0_ (NBFRs) − VEA_T1_ (Solvent)
6	NBFRs_S0_ + Solvent_T1_* → NBFRs·^−^ + Solvent·^+^	ΔG6 = VIE_T1_ (Solvent) − VEA_S0_ (NBFRs)

**Table 2:** Rate constant and half-life and correlation coefficient of PBBA, PBEB, PBT and HBB under three wavelength ranges in n-hexane and in an initial concentration of 1 mg/L. All data were fitted by the pseudo-first-order kinetics equation.

Optical Wavelength (nm)	Rate Coefficient (min^−1^)			
	PBBA	PBEB	PBT	HBB
180~400	0.3008 ± 0.00478	0.1943 ± 0.00123	0.1800 ± 0.00040	0.1702 ± 0.00278
334~365	0.0433 ± 0.00131	0.0289 ± 0.00478	0.0280 ± 0.00461	0.0265 ± 0.00208
400~700	0.0099 ± 0.00118	0.0063 ± 0.00053	0.0058 ± 0.00041	0.0091 ± 0.00151
	Half-life (min)			
180~400	2.31	3.57	3.85	4.07
334~365	16.03	25.06	25.86	26.45
400~700	71.93	112.12	120.4	79.93
	R^2^			
180~400	0.992	0.993	0.993	0.976
334~365	0.967	0.957	0.956	0.991
400~700	0.992	0.979	0.982	0.979

**Table 3:** Rate constant, half-life and correlation coefficient of PBBA, PBEB, PBT and HBB in three initial concentrations in n-hexane under wavelength of 334~365 nm. All data were fitted by the pseudo-first-order kinetics equation.

Initial Concentration (mg/L)	Rate Coefficient (min^−1^)			
	PBBA	PBEB	PBT	HBB
0.25	0.0736 ± 0.00000	0.0513 ± 0.00768	0.0379 ± 0.00004	0.0784 ± 0.00053
0.5	0.0683 ± 0.00363	0.0324 ± 0.00012	0.0307 ± 0.00004	0.0578 ± 0.00029
1	0.0433 ± 0.00131	0.0289 ± 0.00478	0.0280 ± 0.00461	0.0265 ± 0.00208
	Half-life (min)			
0.25	9.42	13.98	18.31	8.85
0.5	10.2	21.43	22.61	12
1	16.03	25.06	25.86	26.45
	R^2^			
0.25	0.99	0.971	0.996	0.984
0.5	0.996	0.997	0.996	0.998
1	0.967	0.957	0.956	0.991

**Table 4:** Rate constant, half-life and correlation coefficient of PBBA, PBEB, PBT and HBB in three initial concentrations in n-hexane under wavelength of 180~400 nm. All data were fitted by the pseudo first-order kinetics equation.

Initial Concentration (mg/L)	Rate Coefficient (min^−1^)			
	PBBA	PBEB	PBT	HBB
0.25	0.2804 ± 0.03584	0.1552 ± 0.00327	0.1477 ± 0.00527	0.1586 ± 0.00265
0.5	0.2885 ± 0.00380	0.1146 ± 0.00131	0.1141 ± 0.00780	0.1173 ± 0.00151
1	0.3008 ± 0.00478	0.1943 ± 0.00123	0.1800 ± 0.00040	0.1702 ± 0.00278
	Half-life (min)			
0.25	2.53	4.47	4.70	4.37
0.5	2.47	6.05	6.12	5.91
1	2.31	3.57	3.85	4.07
	R^2^			
0.25	0.998	0.990	0.980	0.997
0.5	0.980	0.984	0.978	0.982
1	0.992	0.993	0.993	0.976

**Table 5:** Rate constant, half-life and correlation coefficient of PBBA, PBEB, PBT and HBB in three organic solvents in an initial concentration of 1 mg/L under wavelengths of 180~400 nm. All data were fitted by the pseudo-first-order kinetics equation.

Solvent	Rate Coefficient (min^−1^)			
	PBBA	PBEB	PBT	HBB
HEX	0.3008 ± 0.00478	0.1943 ± 0.00123	0.1800 ± 0.00040	0.1702 ± 0.00278
ACE	0.0299 ± 0.00057	0.0170 ± 0.00045	0.0168 ± 0.00065	0.0124 ± 0.00020
TOL	0.0534 ± 0.00069	0.0512 ± 0.00229	0.0469 ± 0.00229	0.0408 ± 0.00131
	Half-life (min)			
HEX	2.31	3.57	3.85	4.07
ACE	23.19	40.7	41.35	56.15
TOL	13	13.58	14.83	17.02
	R^2^			
HEX	0.992	0.993	0.993	0.976
ACE	0.965	0.942	0.934	0.933
TOL	0.994	0.991	0.985	0.986

**Table 6:** Total energy (E), frontier orbital energy levels (E_HOMO_, E_LUMO_) and energy gap (ΔE_gap_) of NBFRs in different solvents.

Compound	Solvents	ε	E (a.u.)	HOMO (eV)	LUMO (eV)	ΔE_gap_ (eV)
HBB	GAS	1.00	−15658.84586	−6.99061	−2.26943	4.72118
	n-hexane	1.58	−15658.86152	−6.98762	−2.27596	4.71165
	toluene	2.37	−15658.86295	−6.98462	−2.27406	4.71057
	acetone	20.70	−15658.84914	−6.95768	−2.25338	4.70431
PBBA	GAS	1.00	−13393.01210	−6.93945	−1.96303	4.97642
	n-hexane	1.58	−13393.03012	−6.92612	−1.96085	4.96526
	toluene	2.37	−13393.03222	−6.92122	−1.95813	4.96309
	acetone	20.70	−13393.03260	−6.89210	−1.93745	4.95465
PBEB	GAS	1.00	−13166.38381	−6.81618	−1.84793	4.96826
	n-hexane	1.58	−13166.39881	−6.79931	−1.84575	4.95356
	toluene	2.37	−13166.40023	−6.79632	−1.84521	4.95111
	acetone	20.70	−13166.39808	−6.77292	−1.83296	4.93996
PBT	GAS	1.00	−13127.06888	−6.82326	−1.85663	4.96662
	n-hexane	1.58	−13127.08355	−6.80503	−1.85201	4.95302
	toluene	2.37	−13127.08493	−6.80149	−1.85065	4.95084
	acetone	20.70	−13127.08302	−6.77292	−1.83459	4.93832

**Table 7:** Vertical transition energies (ET1), vertical ionization energies (VIE) and vertical electron affinities (VIE) for the NBFRs and the solvent molecules (n-hexane; toluene; acetone) (eV).

Compound	Solvents	ET1	VIES0	VEAS0	VIET1	VEAT1
n-hexane	n-hexane	9.5261	8.8109	−2.8572	−0.7152	6.6689
toluene	toluene	3.7512	7.2657	−0.6611	3.5145	3.0901
acetone	acetone	3.9244	7.4011	0.9553	3.4767	4.8797
HBB	n-hexane	3.2317	8.1835	1.2648	4.9483	4.5
	toluene	3.2327	8.0939	1.355	4.8587	4.5902
	acetone	3.2382	6.8619	2.3836	3.6267	5.6188
PBBA	n-hexane	3.3261	8.0651	1.1271	4.7378	4.4544
	toluene	3.3264	7.9897	1.2075	4.6624	4.5348
	acetone	3.3273	7.2988	1.9489	3.9715	5.2762
PBEB	n-hexane	3.3068	7.8546	0.8708	4.5393	4.1861
	toluene	3.3078	7.7696	0.9626	4.4543	4.2779
	acetone	3.3153	7.0433	1.7075	3.728	5.0228
PBT	n-hexane	3.3075	7.8543	0.8802	4.5376	4.1969
	toluene	3.3086	7.7599	0.9766	4.4432	4.2933
	acetone	3.3167	7.0326	1.7193	3.7159	5.036

**Table 8:** Gibbs free energy values from ΔG1 to ΔG6 of the photoinduced electron transfer reactions between the solvent molecules and the NBFRs (eV).

Compound	Solvents	ΔG1	ΔG2	ΔG3	ΔG4	ΔG5	ΔG6
HBB	n-hexane	7.8055	4.3109	−1.7206	−5.2152	1.5146	−1.9800
	toluene	5.5199	2.6755	1.7687	−1.0757	5.0039	2.1595
	acetone	2.6714	1.7824	−1.2530	−2.1420	1.9822	1.0932
PBBA	n-hexane	7.5950	4.3565	−1.9311	−5.1696	1.3962	−1.8423
	toluene	5.3236	2.7310	1.5724	−1.0202	4.8997	2.3071
	acetone	3.0162	2.1250	−0.9082	−1.7994	2.4191	1.5279
PBEB	n-hexane	7.3965	4.6248	−2.1296	−4.9013	1.1857	−1.5860
	toluene	5.1155	2.9878	1.3643	−0.7634	4.6796	2.5519
	acetone	2.7727	2.3784	−1.1517	−1.5460	2.1636	1.7693
PBT	n-hexane	7.3948	4.6140	−2.1313	−4.9121	1.1854	−1.5954
	toluene	5.1043	2.9725	1.3531	−0.7787	4.6698	2.5380
	acetone	2.7606	2.3651	−1.1638	−1.5593	2.1529	1.7574

## Data Availability

Not applicable.

## Supplementary Materials

The following supporting information can be downloaded at: https://www.mdpi.com/article/10.3390/ijerph191811690/s1, Figure S1: Ultraviolet visible absorption spectrum of PBBA, PBEB, PBT and HBB. The absorption spectrum is the theoretical value obtained by Gaussian calculation; Figure S2: Ultraviolet visible absorption spectrum of n-hexane, toluene, acetone; Figure S3: Overall device diagram of photodegradation experiment and schematic diagram of the reactor (a two-layer glass container equipped with a quartz tube for light inside). Table S1: The bond length and dipole moment in gas and four different solvents of NBFRs.
