# Supplementary material for: Photodegradation Kinetics and Solvent Effect of New Brominated Flame Retardants (NBFRS) in Liquid Medium
Source: Int J Environ Res Public Health. 2022 Sep 16;19(18):11690. doi: 10.3390/ijerph191811690 (PMC9517406; doi:10.3390/ijerph191811690)
Supplement: Supplementary file 1 [file ijerph-19-11690-s001.zip › ijerph-1873501-supplementary.pdf]

Table S1 The bond length and dipole moment in gas and four different solvents of NBFRs

Compound	Solvents	e	-C-C- (Å)	-C-Br- (Å)			dipole moment Debye
				ortho-	meta-	para-	
HBB	GAS	1	-	1.893	1.893	1.893	0
	n-hexane	1.58	-	1.895	1.895	1.895	0
	toluene	2.37	-	1.895	1.895	1.895	0
	acetone	20.7	-	1.895	1.895	1.895	0
PBBA	GAS	1	1.528	1.903	1.896	1.899	1.4482
	n-hexane	1.58	1.528	1.904	1.898	1.901	2.8404
	toluene	2.37	1.528	1.904	1.898	1.901	2.9098
	acetone	20.7	1.528	1.905	1.899	1.903	3.3765
PBEB	GAS	1	1.515	1.908	1.895	1.895	1.4189
	n-hexane	1.58	1.515	1.91	1.897	1.897	1.6802
	toluene	2.37	1.515	1.91	1.897	1.897	1.7321
	acetone	20.7	1.514	1.91	1.898	1.898	2.0656
PBT	GAS	1	1.509	1.905	1.894	1.895	1.3587
	n-hexane	1.58	1.508	1.907	1.896	1.897	1.6118
	toluene	2.37	1.507	1.907	1.896	1.897	1.6626
	acetone	20.7	1.507	1.907	1.897	1.898	2.0119

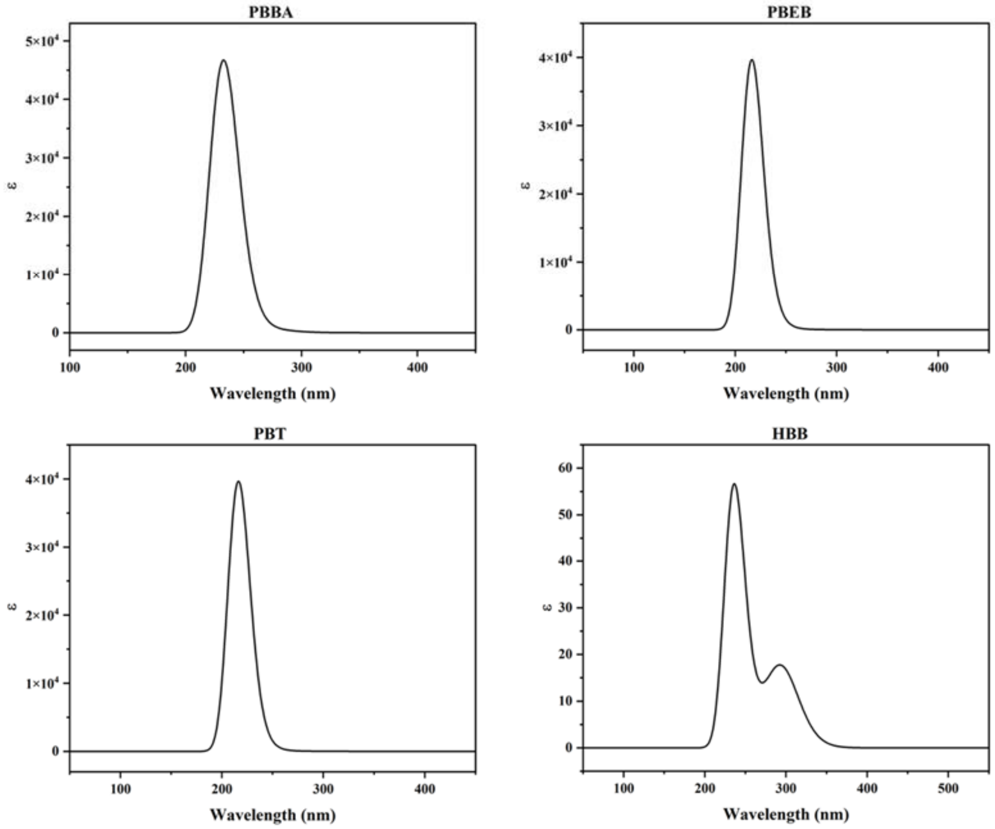

Figure S1. Ultraviolet visible absorption spectrum of PBBA, PBEB, PBT and HBB. The absorption spectrum is the theoretical value obtained by Gaussian calculation.

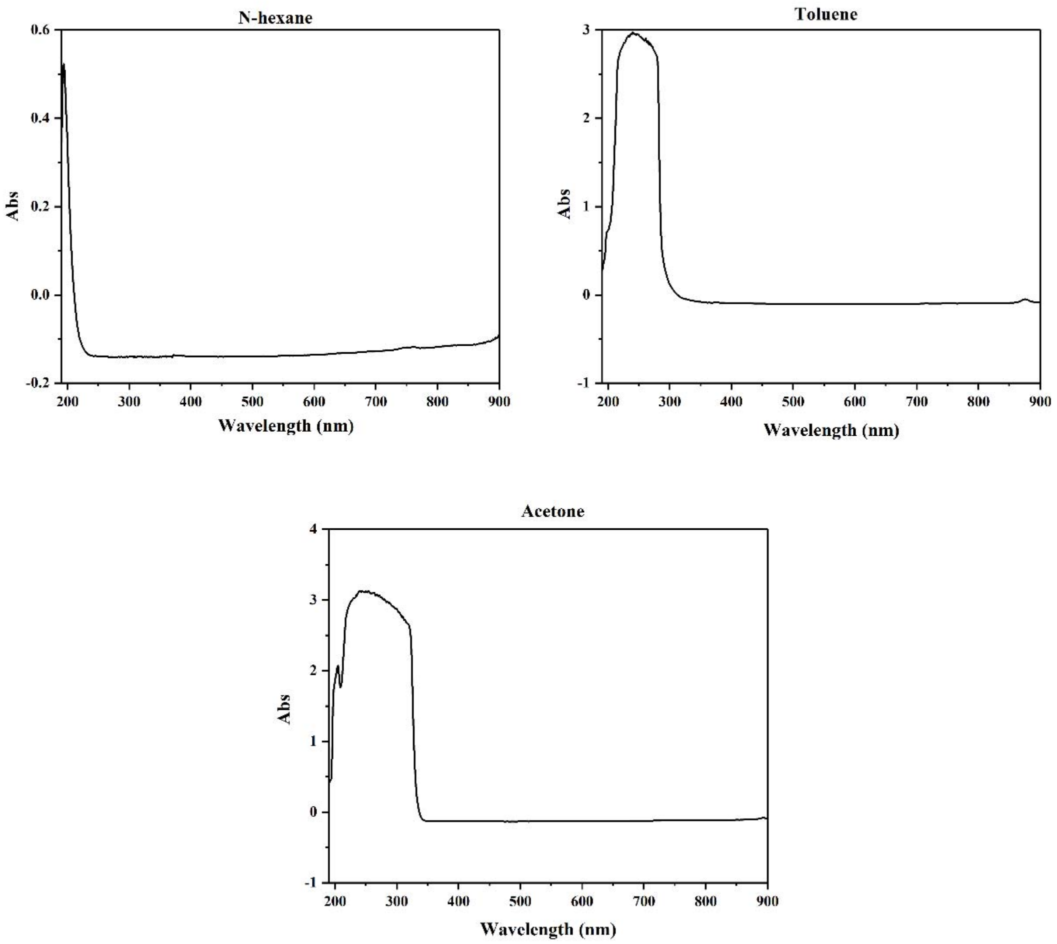

Figure S2 Ultraviolet visible absorption spectrum of n-hexane, toluene, acetone.

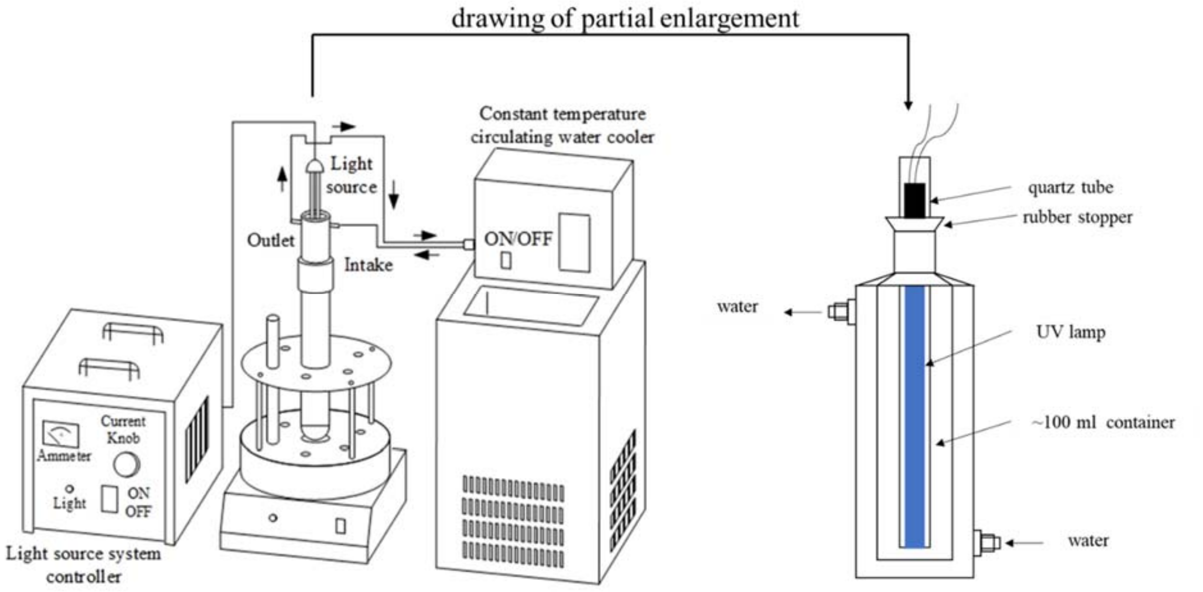

Figure S3. Overall device diagram of photodegradation experiment and schematic diagram of the reactor (a two-layer glass container equipped with a quartz tube for light inside).
